# Neurochemical measurements in the zebrafish brain

**DOI:** 10.3389/fnbeh.2015.00246

**Published:** 2015-09-08

**Authors:** Lauren J. Jones, James E. McCutcheon, Andrew M. J. Young, William H. J. Norton

**Affiliations:** Department of Neuroscience, Psychology and Behaviour, University of LeicesterLeicester, UK

**Keywords:** zebrafish, fast scan cyclic voltammetry, adult brain, dopamine, 5-HT, histamine, pH, neurochemistry

## Abstract

The zebrafish is an ideal model organism for behavioral genetics and neuroscience. The high conservation of genes and neurotransmitter pathways between zebrafish and other vertebrates permits the translation of research between species. Zebrafish behavior can be studied at both larval and adult stages and recent research has begun to establish zebrafish models for human disease. Fast scan cyclic voltammetry (FSCV) is an electrochemical technique that permits the detection of neurotransmitter release and reuptake. In this study we have used *in vitro* FSCV to measure the release of analytes in the adult zebrafish telencephalon. We compare different stimulation methods and present a characterization of neurochemical changes in the wild-type zebrafish brain. This study represents the first FSCV recordings in zebrafish, thus paving the way for neurochemical analysis of the fish brain.

## Introduction

A central goal of neuroscience is to understand how the brain processes stimuli in order to tailor an appropriate behavioral response. Initially, each behavior was thought to be driven by a dedicated neural circuit in the brain (Zupanc and Lamprecht, 2000). However, recent research suggests that discrete behaviors can be produced by the interaction of diffuse neural networks with overlapping functions (Bargmann, 2012). Thus, dramatically different behaviors can be driven by the same neurons acting in parallel circuits. Rather than being hard-wired entities, neural circuits exhibit plasticity due to short-term neuromodulatory activity and longer-term structural reorganization at the synaptic level (Zupanc and Lamprecht, 2000; Bargmann, 2012). Therefore, a combination of approaches combining information from a range of scientific disciplines is needed to identify the network components that drive behavior.

The zebrafish is a powerful model organism for developmental biology and neuroscience. Zebrafish are also an ideal species to investigate the neural circuits that drive behavior since their relative transparency until larval stages permits the visualization and manipulation of neurons within the intact brain (Fetcho and Liu, 1998; Arrenberg and Driever, 2013; Bonan and Norton, 2015; Feierstein et al., 2015). Neural circuits can be mapped using calcium imaging, bioluminescence or electrophysiology to monitor neural activity in freely behaving fish (Higashijima et al., 2003; Naumann et al., 2010). Techniques such as genetic ablation or optogenetics can then be used to functionally connect circuits to behavior (Nagel et al., 2003; Curado et al., 2007; Zhang et al., 2007; Del Bene and Wyart, 2012). An alternative approach, based upon forward genetics, is to identify mutant lines that exhibit interesting behavioral phenotypes. The expression profile of the mutated loci can be used as a starting point to examine the alterations to brain structure and function that underpin aberrant behavior (Webb et al., 2009; Norton et al., 2011; Ziv et al., 2013).

Previous studies have used quantitative PCR, immunohistochemistry and electrophysiology to investigate the function of the zebrafish brain. In our laboratory we complement these approaches by using fast-scan cyclic voltammetry (FSCV) to quantify the release- and reuptake of neurotransmitters at the synapse on a sub-second time-scale (Stamford, 1990; Heien et al., 2004). A voltage waveform is applied to a carbon fiber microelectrode causing oxidation and reduction of electroactive compounds at the surface of the electrode (Stamford, 1990; John and Jones, 2007a). These oxidation and reduction reactions lead to changes in electrical current which are proportional to the concentration of the compound being measured. This data can be visualized as a color plot where current is encoded using a false color scheme and plotted against both applied electrical potential (E_app_) and time (Heien et al., 2004). Typically, *in vitro* neurotransmitter release is evoked by using either electrical stimulation or bath application of a high concentration of potassium. Different analytes, including many neurotransmitters, can be identified on the basis of their voltammograms (a plot of applied voltage against current) using attributes such as the position, shape and relative amplitude of the oxidation and reduction peaks (Stamford, 1990; Heien et al., 2004). Further specificity can be achieved by placing the recording electrode into brain areas containing a single neurotransmitter or by applying specific drugs that modify neurotransmitter reuptake (Dankoski and Wightman, 2013). Finally, if stimulation evokes the release of several analytes, the overlapping voltammograms can be separated by principal component analysis (Heien et al., 2004).

In this study we present a method for FSCV recordings in sagittal slices of the adult zebrafish brain and characterize the release of analytes in the telencephalon. This research provides a basis for examination of zebrafish mutants that display intriguing behavioral phenotypes by uncovering alterations to the dynamics of neurotransmitter release in the brain.

## Materials and methods

### Fish stocks

All experiments were performed on adults of the AB/AB wild-type strain. Standard fish keeping protocols and conditions were followed (Westerfield, 1995). Fish were anesthetized in MS333 and culled by decapitation (a Schedule 1 procedure under the Animals (Scientific Procedures) Act 1986 Amendment Regulations 2012). All protocols are covered by appropriate personal licenses: PIL 60/13671 (WHJN) and PIL I98F58CD7 (LJJ).

### FSCV equipment

**Figure 1:**
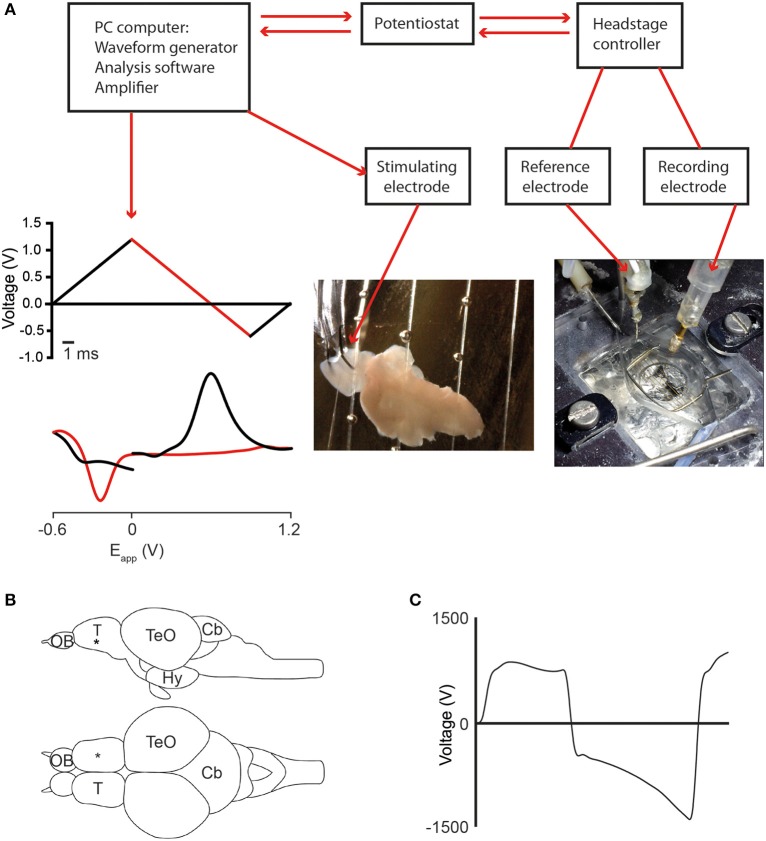
**Fast scan cyclic voltammetry (FCSV) setup and position of the stimulating- and recording electrodes**. **(A)** Diagram showing components of the FSCV setup and the position of the recording electrode in the adult zebrafish brain. The applied voltage waveform (top graph) and a representative cyclic voltammogram for dopamine (lower graph) are also shown, with the forward scan in black and the reverse scan in red. **(B)** Schematic representation showing a lateral and dorsal view of the adult zebrafish brain. The black asterisk marks the position of the recording electrode in the telencephalon. **(C)** Diagram showing characteristic non-Faradaic background signal that is subtracted to generate the background-subtracted voltammograms shown throughout the paper. Abbreviations: Cb, cerebellum; Hy, hypothalamus; OB, olfactory bulb; T, telencephalon; TeO, optic tectum.

The setup for FSCV was custom built and consists of a tissue bath, stimulating electrode, recording and reference electrodes connected to a computer and amplifier (Figure 1A). Carbon fiber microelectrodes (tip size 7 × 120 μm) were used as recording electrodes and an Ag/AgCl electrode as a reference. The recording and reference electrodes were connected to a potentiostat and headstage circuit (ChemClamp, Dagan Instruments, USA) and a computer running TarHeel (Chapel Hill, University of North Carolina) voltammetry software. The waveform (Figure 1A) was applied at 10 Hz.

### Electrode manufacture

Glass capillary electrodes were used for all our FSCV experiments. Electrodes were manufactured as described in Fortin et al. (2015). A single carbon fiber was aspirated into a borosilicate glass capillary (World Precision Instruments, 100 mm length, 1/0.58 mm OD/ID). The glass was pulled to a fine tip using a vertical needle puller (PE-21, Narishige) and the exposed carbon fiber was cut to a length of 100 μm using a scalpel. A wire coated in silver conductive paint (Coating Silver Print II, GC Electronics) was inserted into the capillary, secured with a gold pin (Newark) and heat shrink-wrapped to the capillary (FP-301, 3M). In each case, electrodes were tested to ensure a suitable background (non-Faradaic) current profile by applying the voltage waveform (see below) at 60 Hz. If the current signal was adequate the electrode was cycled at 60 Hz for a minimum of 15 min to reduce background drift.

### Voltage input waveforms

The voltage input waveform was scanned at a rate of 400 V/s in the following pattern: 0 V → +1.2 V → −0.6 V → 0 V (John and Jones, 2007a). With this waveform, dopamine oxidizes at ~ +0.6 V and shows one reduction peak at ~ −0.2 V; 5-HT oxidizes at ~ +0.6 V and shows two reduction peaks at ~0 and ~ −0.5 V; and histamine shows two reduction peaks on the forward scans of the waveform at ~+0.25 and ~ −0.4 V and an oxidation peak on the reverse scan at ~ +1.05 V.

### Flow cell experiments

Flow cell experiments were performed in a custom-built Y-shaped chamber (University of Illinois at Chicago Biology Workshop, Sinkala et al., 2012). The cell permits a carbon-fiber electrode to be exposed to known concentrations of neurotransmitter solutions. All analytes were dissolved in artificial fish cerebrospinal fluid (aCSF) and delivered by gravity perfusion at a rate of 1 ml per min. aCSF (pH 7.4) contained (in mM) 131 NaCl, 2 KCL, 1.25 KH_2_PO_4_, 2 MgSO_4_, 20 NaHCO_3_, 2.5 CaCl, and 10 glucose (Vargas et al., 2011). For some experiments, we prepared aCSF containing 20 mM 4-(2-hydroxyethyl)-1-piperazineethanesulphonic acid (HEPES) adjusted to pH 7.4. The voltage waveform was applied to electrodes at 60 Hz for 15 min prior to the start of each experiment to precondition the electrode (Fortin et al., 2015). Known concentrations of dopamine, histamine, 5-HT, and pH-adjusted aCSF were perfused for 5 s and changes in current were recorded.

### Preparation of tissue for FSCV

Following decapitation, the brain was manually dissected from the skull in a Petri dish containing ice-cold aCSF. Brains were sliced sagittally and transferred to a tube containing additional ice-cold aCSF. Sections were mounted in an organ bath and perfused with oxygenated aCSF (constantly bubbled with 95% O_2_ and 5% CO_2_ and warmed to 32°C by a Peltier heater) at a rate of 1.5 ml per min. Tissue sections were allowed to equilibrate in warmed aCSF for 10–15 min before recording began. The flow rate was regulated by a gravity flow system containing an intravenous dial flow-regulator (World Precision Instruments) and waste aCSF was aspirated using a Dymax 5 suction pump (Charles Austen pumps and RS Components). A micromanipulator (R.C-2R adjustable clamp, Narishige) was used to insert the electrodes into the telencephalon at a depth of approximately 150 μm (Figure 1B).

### Stimulation of neurotransmitter release

Neurotransmitter release was evoked by either bath application of a high concentration of potassium or electrical stimulation. Potassium-evoked release was performed by perfusing tissue with high K^+^ (100 mM, replacing an equimolar amount of NaCl) aCSF for 1 min once a stable 30 s baseline recording had been obtained. Electrically-evoked release was performed with a bipolar stimulating electrode placed close to the carbon fiber recording electrode within the dorsal telencephalon. Current pulses were generated by the acquisition software and applied via a stimulus isolator (Iso-Flex; AMP Instruments). The tissue was allowed to recover for a minimum of either 5 min (electrical stimulation) or 30 min (for high K^+^ stimulation) between stimulations. In some experiments we added drugs targeting neurotransmitter systems to the aCSF: 10 μM GBR 12909 (selective DA reuptake inhibitor; Sigma Aldrich D052); or 10 μM cocaine hydrochloride (DA, NA and 5-HT reuptake inhibitor; Sigma Aldrich C5776). GBR 12909 was first dissolved in DMSO with gentle warming before being directly added to the aCSF. Cocaine was made into a stock solution in water before being added to aCSF. Drugs were not perfused onto tissue until at least 3 stable baseline recordings had been obtained.

### Fast-scan cyclic voltammetry procedure

Voltage waveforms were applied to electrodes using TarHeel software and the resulting changes to current were recorded and analyzed. Carbon fiber microelectrodes generate a characteristic background signal that can be subtracted to yield the Faradaic current caused by oxidation and reduction of compounds (Figure 1C, Baur et al., 1988; John and Jones, 2007a). Neurotransmitters were identified upon the basis of their cyclic voltammograms (noting the position and height of oxidation and reduction peaks) and color plots permitted the visualization of release dynamics over time.

### Statistical analyses

The percentage of variance in experimental data (voltammograms recorded in the telencephalon) accounted for by template data (voltammograms generated in a flow cell) was assessed using the CV match programme in TarHeel (Robinson et al., 2003). Analyses were only performed on experimental data obtained using the same electrode as that used to collect template data. We reported the values as *r*^2^, which was deemed to be significant when exceeding a threshold of *r*^2^ = 0.75 as described in Heien et al. (2003). Principal component analysis was performed in TarHeel (Heien et al., 2004, 2005; Keithley and Wightman, 2011). An *in vitro* training set composed of cyclic voltammograms for dopamine, 5-HT, histamine and both acidic and basic pH shifts and combinations of these four factors were used for the final analysis (Heien et al., 2004, 2005). At least five voltammograms for each species (neurotransmitter/pH change) or combination were included. Training sets were deemed to fit the data appropriately if the maximum value shown on the Q plot did not exceed the threshold Qa value (686681). Current vs. time data recorded following application of neurotransmitter reuptake inhibitors to the tissue were extracted from TarHeel software and imported into Clampfit 10.2 (part of pCLAMP 10.2 software package; Molecular Devices). Baseline correction was applied to all recordings to account for increasing/decreasing baselines and electrode drift thus ensuring a stable flat baseline for analysis. Clampfit was also used for peak analysis, providing values for peak amplitude and multiple time parameters reflective of reuptake. This included half width (the time taken for the peak to reach- and return to half peak amplitude), T half (time taken to decay to half peak amplitude from peak), tau decay (a time constant representing decay of current), and peak area (nA^*^s) all of which have been deemed appropriate measures of neurotransmitter reuptake (Yorgason et al., 2011). Statistical analyses were conducted using GraphPad Prism 6 for Windows. One-Way repeated measures ANOVA tests were performed for all measures, with time as the repeated measures variable. Control values were obtained by averaging values from three control stimulations prior to drug perfusion. When statistical significance was indicated (*p* < 0.05), *post-hoc* analyses were conducted using Dunnett's multiple comparisons test, comparing each time-point to control and adjusting *p*-values accordingly. Non-parametric Friedman tests were used when data were not normally distributed. When statistical significance was indicated, *post-hoc* analyses were conducted using Dunn's multiple comparisons test adjusting *p*-values accordingly.

## Results

### Characterization of neurotransmitter profiles in a flow cell

**Figure 2:**
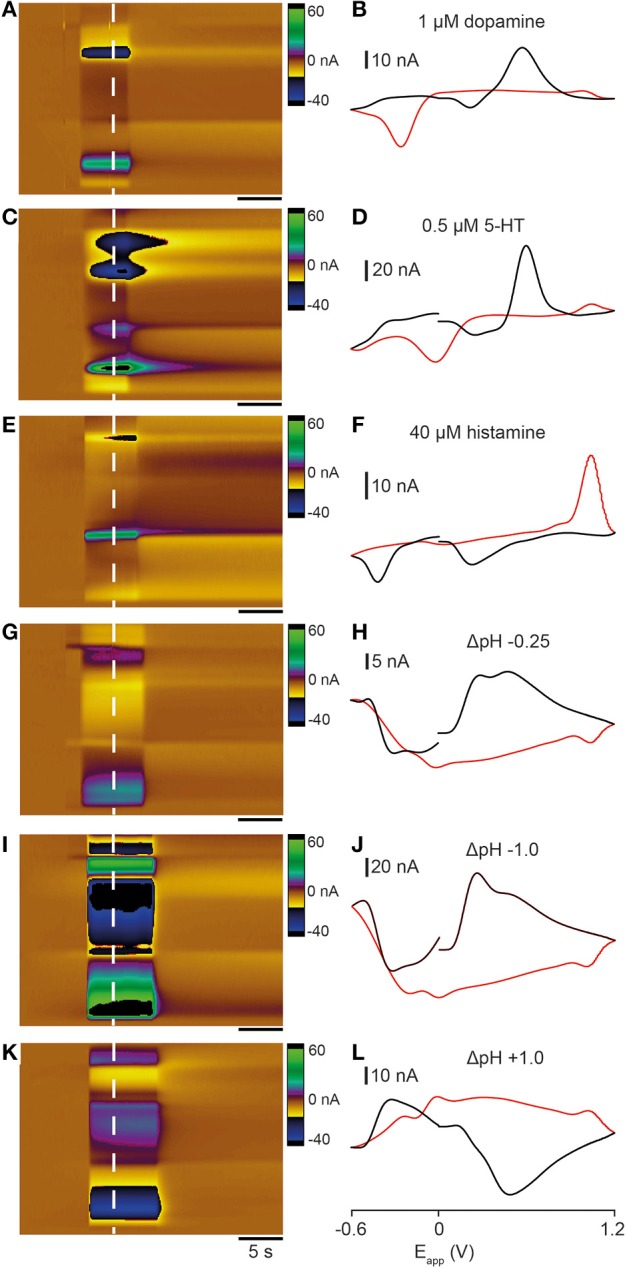
**Comparison of cyclic voltammograms generated by exposing electrodes to dopamine, 5-HT, histamine, and pH changes in a flow cell**. Color plots **(A,C,E,G,I,K)** and cyclic voltammograms (**B,D,F,H,J,L**; black lines represent forward scan and red lines reverse scan) taken at the time point indicated by the dashed white lines. **(A,B)** 1 μM dopamine solution. **(C,D)** 0.5 μM 5-HT solution. **(E,F)** 40 μM histamine solution. **(G,H)** −0.25 units acidic pH change (pH 7.4 → pH 7.15). **(I,J)** -1.0 units acidic pH change (pH 7.4 → pH 6.4). **(K,L)** +1.0 units basic pH change (pH 7.4 → pH 7.4 → pH 8.4). Scale bar in **(A,C,E,G,I,K)** represents 5 s.

**Figure 3:**
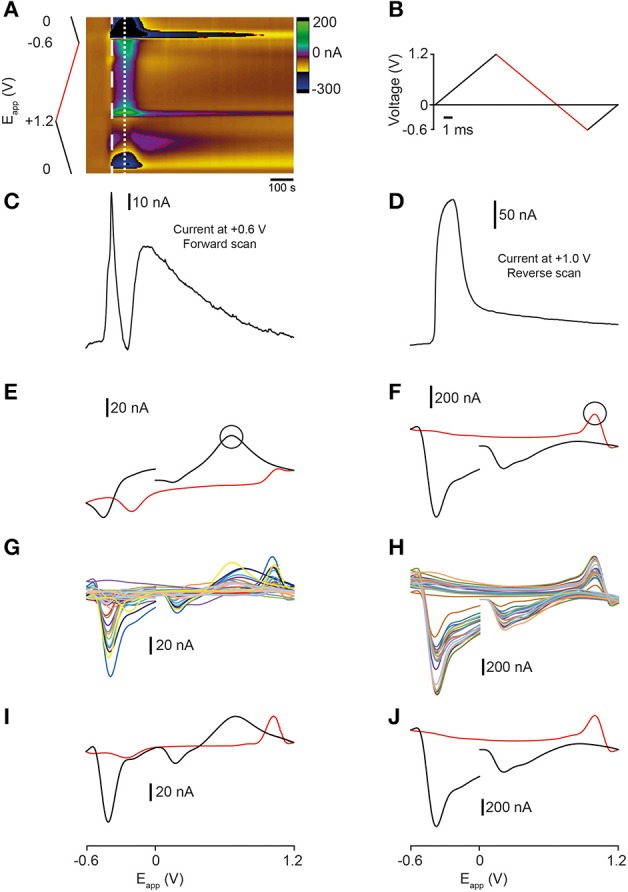
**Comparison of analytes evoked by high K^+^ aCSF stimulation of the zebrafish telencephalon**. **(A)** Color plot showing changes in current following stimulation with high K^+^ aCSF. **(B)** Applied waveform ranging from 0 V → +1.2 V → −0.6 V → 0 V (the holding potential). The forward scan of the waveform is colored black and the reverse scan is red. **(C)** Current vs. time plot showing the profile of current changes at *E*_app_ = ~ +0.6 V, the point in the waveform indicated by the black circle in **(E)**. **(D)** Current vs. time plot showing the profile of current changes at *E*_app_ = ~ +1.0 V on the reverse scan of the waveform, the point indicated by the circle in **(F)**. **(E)** Representative cyclic voltammogram taken at the time point indicated by the thick dashed white line in **(A)**. **(F)** Representative cyclic voltammogram taken at the time point indicated by the thin dashed white line in **(A). (G)** Cyclic voltammograms from 24 separate stimulations taken at the time point indicated by the thick dashed white line in **(A)**. **(H)** Cyclic voltammograms from 20 independent experiments taken at the time point indicated by the thin dashed white line in **(A)**. **(I)** Average cyclic voltammogram derived from data presented in **(G)**. **(J)** Average cyclic voltammogram derived from data presented in **(H)**.

As a first step toward characterizing analyte release in zebrafish we collected template FSCV data for dopamine, 5-HT and histamine, neurotransmitter systems that send extensive projections to the telencephalon. We used a flow cell (Sinkala et al., 2012), a microfluidic device that permits electrodes to be exposed to standard neurotransmitter solutions, to collect representative color plots and cyclic voltammograms. We exposed carbon fiber electrodes to a known concentration of each neurotransmitter or pH shift (using the voltage waveform shown in **Figure 3B**). Application of 1 μM dopamine produced an increase in current at ~ +0.6 V and a reduction peak at ~ −0.25 V (Figures 2A,B). A 0.5 μM 5-HT solution also produced an increase in current ~ +0.6 V but reduction peaks occurred at ~ 0 and ~ −0.5 V (Figures 2C,D). Application of 40 μM histamine produced a very different response, with an increase in current on the reverse scan at ~ +1.0 V and two reduction peaks on the forward scan at ~ +0.25 and ~ −0.4 V (Figures 2E,F). We also investigated the effect of altering pH on the oxidation potentials of voltammograms. An acidic change of −0.25 pH units (i.e., pH 7.15) produced an oxidation peak at ~ +0.5 V and reduction peaks at both ~ +1.1 and ~ −0.3 V (Figures 2G,H). An acidic change of −1.0 pH units (i.e., pH 6.4) produced a similar voltammogram with a sharp oxidation peak at ~ +0.5 V and reduction peaks at both ~ +1.1 and ~ −0.3 V (Figures 2I,J). A basic pH change of +1.0 units (i.e., pH 8.4) produced a large oxidation peak on the reverse scan at ~ +1.1 V and reduction peaks on the forward scans at ~ +0.4 and ~ −0.5 V, similar to the neurotransmitter histamine (Figures 2K,L). Together, these experiments demonstrate the characteristic shapes of cyclic voltammograms that are produced by exposing electrodes to neurotransmitter solutions and changes in pH.

### Fast-scan cyclic voltammetry in the adult zebrafish brain

We next investigated whether fast-scan cyclic voltammetry (FSCV) could be used to measure the release of analytes in sagittal sections of the adult zebrafish brain. The zebrafish dorsal telencephalon receives extensive 5-HT-positive projections from the raphe- and pretectal nuclei (Lillesaar et al., 2009). We therefore applied a voltage waveform optimized for measurements of 5-HT (John and Jones, 2007a) and depolarised neurons and terminals with aCSF containing a high concentration of K^+^ (100 mM K^+^; hereafter high K^+^ aCSF). Bath application of high K^+^ aCSF led to changes in current at several points in the voltage waveform. A cyclic voltammogram extracted at ~10 s after stimulation displays characteristics that could reflect the oxidation of dopamine and/or 5-HT, including a prominent peak in current on the forward scan at ~ +0.6 V (Figure 3E). A rapid increase in oxidative current is observed at the point in the waveform that corresponds to the peak of this signal (~ +0.6 V; Figure 3C). However, this current vs. time plot also exhibits a striking dip in current which is most likely due to the decrease in current at around ~ +0.2 V masking the oxidation peak at ~ +0.6 V.

The cyclic voltammogram extracted 30 s after application of high K^+^ aCSF displays a different set of characteristics that are suggestive of an additional compound being oxidized on the reverse scan of the waveform (Figure 3F). These features include a dip in current at ~ +0.6 V, a large increase in current at around ~ +1.0 V and two decreases in current at ~ +0.2 and ~ −0.4 V suggesting that reduction occurs on the forward scan of the waveform (Figures 3D,F). Examination of the current vs. time plot at +1.0 V shows a large increase in current that returns toward baseline upon washout of the high K^+^ aCSF (Figure 3D). The cyclic voltammogram of the second analyte exhibited characteristics of the voltammogram for the neurotransmitter histamine (Figure 2F) which has distinct characteristics including a reduction peak on the forward scan and an oxidation peak on the reverse scan (in contrast to the oxidation and reduction profile of dopamine (Figure 2B) and 5-HT (Figure 2D) (Pihel et al., 1995; Hashemi et al., 2011; Chang et al., 2012). Interestingly, both current vs. time plots reveal a long time-course of release with analytes failing to return fully to pre-stimulation baseline levels. We next compared FSCV data obtained from multiple independent experiments (*n* = 12 sagittal sections from eight fish for data extracted at ~10 s, and *n* = 12 sagittal sections from eight fish for data extracted at ~30 s). The shape of the voltammograms that we obtained was highly reproducible, both within- and across-experiments (Figures 3G,H). Furthermore, average voltammograms compiled from these experiments looked similar to representative recordings in the telencephalon, with oxidation- and reduction peaks occurring at the same potentials (compare Figures 3I–E and Figures 3J–F). This suggests that we can compare FSCV data across different animals or mutant lines.

### Electrically-evoked neurotransmitter release in the zebrafish telencephalon

**Figure 4:**
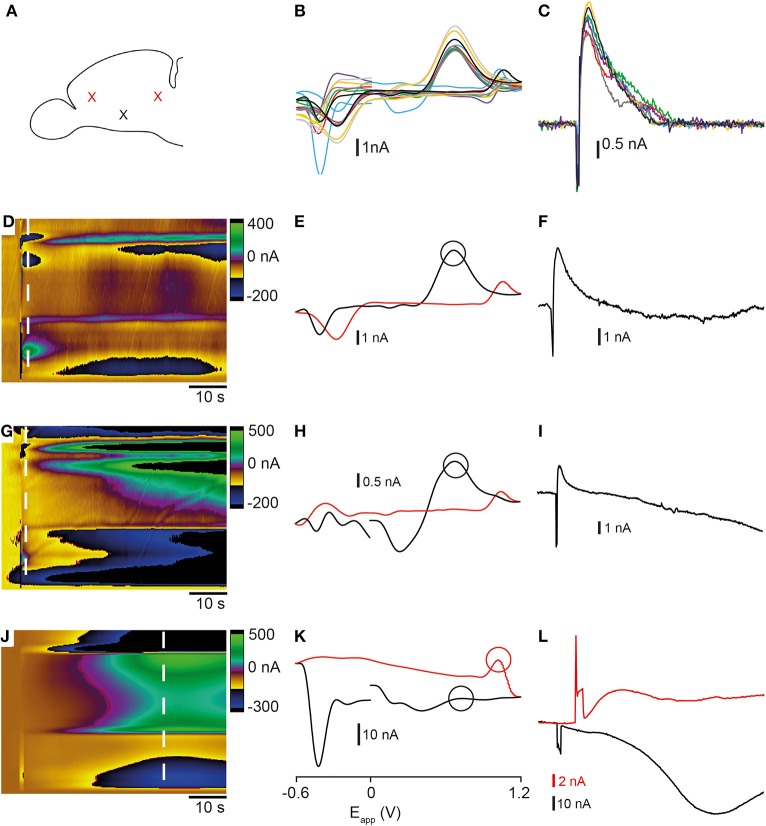
**Comparison of electrically-stimulated release of analytes in the telencephalon**. **(A)** Schematic representation showing lateral view of zebrafish telencephalon. The red crosses show the position of tips of the stimulating electrode and the black cross shows the position of the recording electrode used in these experiments. **(B,C)** Voltammograms and current vs. time plots from eight repeated stimulations in one slice using optimal parameters [20 pulses (pulse width of 4 ms) and a voltage of 500 μA, 60 Hz]. **(D–F)** Color plot, voltammogram, and current vs. time plot from a single representative experiment using optimal stimulation. **(G–I)** Color plot, voltammogram and current vs. time plot from a single representative experiment using low intensity stimulation parameters [20 pulses (pulse width of 4 ms), and a voltage of 300 μA, 60 Hz]. **(J–L)** Color plot and voltammogram and current vs. time plots from a single representative experiment using high intensity stimulation parameters [60 pulses (pulse width of 4 ms), voltage of 1 mA, 60 Hz]. High intensity stimulation led to the release of analytes at two different points in the voltammogram [black and red lines in **(L)**]. **(D,G,J)** Dashed lines show the position at which the voltammograms were extracted and black and red circles depict points at which current vs. time plots were taken.

Bath application of high K^+^ aCSF produced a current vs. time plot with a prolonged release profile that did not return to pre-stimulation baseline levels. In order to clarify whether this was an artifact caused by electrode drift during the long time necessary for complete washout to occur, we used electrical stimulation to evoke the release of analytes (Figure 4A). Electrical stimulation of local terminals using optimal parameters (20 pulses with a pulse width of 4 ms, 60 Hz, 500 μA) resulted in an increase in current on the forward part of the waveform (Figures 4C,F) that rapidly returned to baseline (*n* = 8 stimulations from a single sagittal section in Figures 4B,C). It also produced a cyclic voltammogram with a shape similar to that obtained using high K^+^ aCSF suggesting that both types of stimulation evoke the release of a similar mixture of analytes (Figures 4B,D,E). We tested this possibility using the CV match algorithm in TarHeel. Comparison of an example electrical stimulation with an example K^+^ stimulation gave an *r*^2^-value of 0.876, indicating that both types of stimulation evoke similar neurochemical changes in the tissue. However, the oxidation peak at ~ +0.6 V and reduction peak at ~ −0.2 V were much more prominent when using electrical stimulation than in voltammograms obtained using high K^+^ aCSF. The lowest intensity stimulation that we could use to trigger analyte release in the telencephalon was 20 pulses with a pulse width of 4 ms, 60 Hz, 300 μA. This produced a voltammogram with an oxidation peak at ~ +0.6 V and a smaller reduction peak at ~0.2 V (Figures 4G–I). In contrast to this, high intensity stimulation (1 mA, 60 Hz, 60 pulses, pulse width 4 ms) produced a cyclic voltammogram with a similar shape to that extracted ~30 s after K^+^ stimulation (Figures 4J,K) with a small oxidation peak occurring on the reverse scan at ~1.1 V and a large reduction peak at ~ −0.4 V). Furthermore, the change in current ~ +0.6 V showed a large decrease similar to the dip in current observed following K^+^ stimulation (Figure 4L). The prolonged time-course of alterations in current suggested that an artifact such as a change in pH had occurred. This indicates that stimulation parameters are an important consideration when attempting to obtain reproducible measurements of neurotransmitter release that are not masked by pH shifts or electrode drift.

### Analysis of the effects of pH changes on current following stimulation of the telencephalon

**Figure 5:**
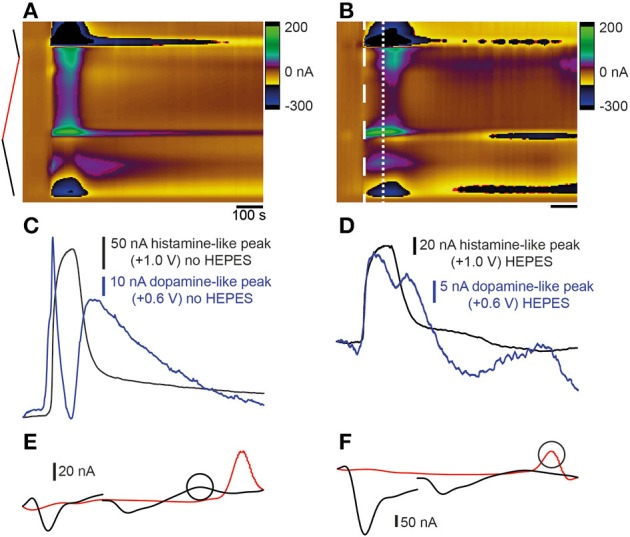
**Comparison of stimulation with high K^+^ aCSF with- and without HEPES buffer**. **(A)** Color plot showing changes in current evoked by stimulation with high K^+^ aCSF without HEPES. The same color plot is shown in Figure 2A. **(B)** Color plot showing changes in current evoked by stimulation with high K^+^ aCSF containing HEPES buffer. Thick dashed white line shows the time point and potential at which the first analyte was maximal. Thin dashed white line shows the time point at which the second analyte was observed. Current vs. time plots for responses obtained in high K+ aCSF without HEPES **(C)** (same traces as shown in Figures 2C,E for comparison) compared to aCSF containing HEPES buffer **(D)**. Blue lines show dopamine-like current and black lines show histamine-like current. **(E)** Voltammogram extracted at time point indicated by thick dashed white line in **(B)**. Circle corresponds to blue line in **(D)**. **(F)** Voltammogram extracted at time point indicated by thin white line in **(B)**. Circle corresponds to black line in **(D)**.

We investigated whether a shift in pH could be contributing to the changes in the current that we measured by adding HEPES buffer to the aCSF. Stimulation using high K^+^ HEPES-buffered aCSF altered the release profile of analytes occurring at the oxidation and reduction potentials for both dopamine and histamine. The large dip normally present at ~10 s after application of high K^+^ aCSF was reduced (compare Figure 5A and Figure 5C with Figure 5B and Figure 5D). Furthermore, addition of HEPES caused the current to return to baseline following stimulation (Figure 5D) suggesting a less prominent shift in background signal. The resulting voltammogram, taken ~10 s after stimulation, (Figure 5E) no longer showed a large reduction in signal at around +0.2 V which seemed to mask the oxidation peak at +0.6 V in previous experiments (compare to Figures 3A,C). A second cyclic voltammogram taken ~30 s after stimulation showed a small increase in current observed at ~ +0.7 V (Figure 5F).

### Combinations of neurotransmitter solutions in the flow cell

**Figure 6:**
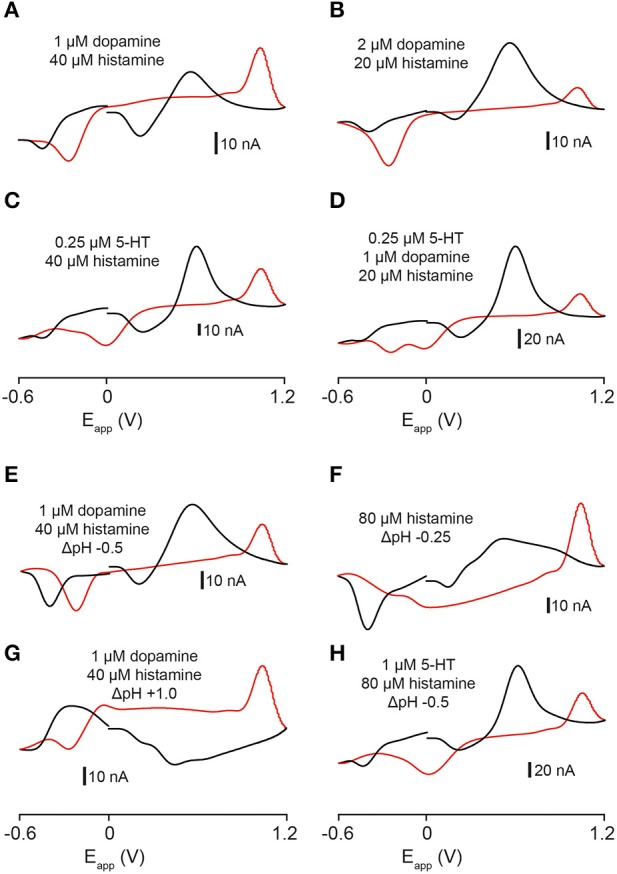
**Comparison of cyclic voltammograms generated by exposing electrodes to mixtures of dopamine, 5-HT, and histamine and pH changes in a flow cell**. **(A)** 1 μM dopamine and 40 μM histamine solutions. **(B)** 2 μM dopamine and 20 μM histamine solutions. **(C)** 0.25 μM 5-HT and 40 μM histamine solutions. **(D)** 0.25 μM 5-HT, 1μM dopamine and 20 μM histamine solutions. **(E)** Voltammogram resulting from exposing an electrode in a flow cell to 1 μM dopamine and 40 μM histamine in the presence of an acidic pH shift (−0.5 pH units, pH 7.4 → pH 6.9). **(F)** Voltammogram resulting from exposing an electrode in a flow cell to 80 μM histamine in the presence of an acidic pH shift (−0.25 pH units; pH 7.4 → pH 7.15). **(G)** Voltammogram resulting from exposing an electrode in a flow cell to 1 μM dopamine and 40 μM histamine in the presence of a basic pH shift (+1.0 pH units, pH 7.4 → pH 8.4). **(H)** Voltammogram resulting from exposing an electrode in a flow cell to 1 μM 5-HT and 80 μM histamine in the presence of an acidic pH shift (−0.5 pH units, pH 7.4 → pH 6.9). Black lines represent forward scan and red lines reverse scan.

The color- and voltage-plots that we obtained from recordings in the zebrafish telencephalon appeared to be influenced by the release of more than one analyte. We applied combinations of dopamine, 5-HT and histamine and pH changes to the electrode in the flow cell and measured changes in current. A combination of 1 μM dopamine and 40 μM histamine produced a cyclic voltammogram with a small oxidation peak at ~ +0.6 V, a larger oxidation peak at around +1.0 V and three reduction peaks at ~ +0.25, ~ −0.2, and ~ −0.4 V (Figure 6A). A combination of 2 μM dopamine and 20 μM histamine produced a very large oxidation peak at ~ +0.6 V, a second oxidation peak on the reverse scan at ~ +1.0 V and reduction peaks at ~ +0.2, ~ −0.2, and ~ −0.4 V (Figure 6B). Likewise, combining 5-HT and histamine produced a voltammogram with a similar oxidation profile but different reduction profile. A mixture of 0.25 μM 5-HT and 40 μM histamine led to oxidation peaks at ~ +0.6 and ~ +1.0 V and reduction peaks at ~ +0.3, ~0, and ~-0.4 V (Figure 6C). We also examined the current changes produced by mixing all three neurotransmitters. A combination of 0.25 μM 5-HT, 1 μM dopamine and 20 μM histamine produced a current plot with oxidation peaks at ~ +0.6 and ~ +1.0 V and four reduction peaks at ~ +0.2, ~0, ~ −0.2, and ~ −0.4 V (Figure 6D). Together, these data indicate that it should be possible to separate signals composed of these three neurotransmitters, since each individual peak is neither inflated nor altered by the presence of a second compound, apart from the overlapping oxidation peak at ~ +0.6 V for dopamine and 5-HT. However, 5-HT can still be identified by its unique reduction peaks, permitting the visual dissociation of these two transmitters. We explored the possibility that pH changes could be influencing the signals that we recorded in the telencephalon by altering the pH of dopamine, histamine and 5-HT mixtures in a flow cell. A combination of -0.5 pH units, 1 μM dopamine and 40 μM histamine (Figure 6E) produced a cyclic voltammogram similar to the voltammogram obtained ~10 s after high K^+^ aCSF stimulation (Figure 2D). Altering the pH of histamine alone (80 μM histamine and −0.25 pH units; Figure 6F) provided a good *in-vitro* representation of the voltammogram for the second analyte obtained with high K^+^ HEPES-buffered aCSF (Figures 6E,F). Despite not being completely identical, it showed changes in current at ~ +0.2, ~ +1.0, and ~ −0.4 V. A basic change in pH (+1.0 units) coupled to 1 μM dopamine and 40 μM histamine produced a voltammogram with a broad reduction peak around +0.4 V (Figure 6G) but no large reduction peak at ~ −0.4 V (Figures 5E,F) suggesting that the pH change was not likely to be basic. Furthermore, addition of 5-HT to the flow cell mixture also produced a voltammogram with a very different shape (1 μM 5-HT, 80 μM histamine and −0.5 pH units) suggesting that 5-HT was unlikely to have contributed to the analytes measured in the telencephalon (Figure 6H).

### CV match analysis of *in vitro* stimulation data

**Figure 7:**
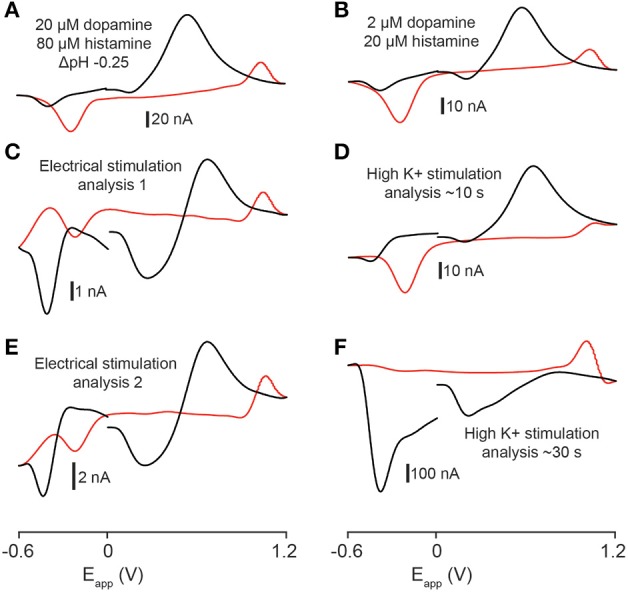
**Statistical analysis of stimulated release. (A)** Template voltammogram resulting from exposing an electrode in a flow cell to 2 μM dopamine and 80 μM histamine in the presence of an acidic pH shift (−0.25 pH units, pH 7.4 → pH 7.15). **(B)** Template voltammogram resulting from exposing an electrode in a flow cell to 2 μM dopamine and 20 μM histamine. **(C–F)** Cyclic voltammograms obtained by either electrical- **(C,E)** or high K^+^ aCSF stimulation **(D,F)** of the telencephalon. The *r*^2^-value for **(A,C)** is 0.846; the *r*^2^-value for **(A,E)** is 0.833; the *r*^2^-value for **(B,D)** is 0.857 and the *r*^2^-value for **(B,F)** is 0.416.

In order to compare the similarity of experimental recordings in tissue to template flow cell data we used the CV match function in TarHeel. We calculated *r*^2^-values between experimental cyclic voltammograms from the telencephalon obtained using optimal stimulation parameters and multiple template cyclic voltammograms (Robinson and Wightman, 2007). Comparison of voltammograms produced by electrical stimulation (four experiments from two sagittal sections taken from two fish) to a mixture of 2 μM dopamine, 80 μM histamine and an acidic pH shift of −0.25 units produced *r*^2^-values that exceeded the accepted threshold of 0.75 in 3 out of 4 stimulations analyzed (for example, Figures 7A,C, *r*^2^ = 0.846; Figures 7A,E, *r*^2^ = 0.833; for further values see Table 1). In one case, a template of 4 μM dopamine, 160 μM histamine and an acidic pH shift of −0.25 units produced the highest *r*^2^-value of 0.878 (see Table 1). High K^+^ aCSF stimulation of the telencephalon produced a voltammogram at ~10 s following stimulation, which we also compared to multiple template cyclic voltammograms (two experiments from two fish) that were significantly similar to a combination of 2 μM dopamine and 20 μM histamine (Figures 7B,D, *r*^2^ = 0.857; for further values see Table 1). However, it was not possible to produce a template voltammogram in the flow cell that gave a significant match to a voltammogram extracted at ~30 s following high K^+^ aCSF stimulation. In summary, the analytes released by electrical stimulation of the zebrafish forebrain are significantly similar to a combination of dopamine, histamine and an acidic pH change of −0.25 units, whereas the analytes released ~10 s after high K^+^ stimulation are indicated to be more similar to a combination of dopamine and histamine.

**Table 1:** **Output for CV Match linear regression analysis comparing template cyclic voltammograms obtained using a flow cell with cyclic voltammograms extracted from experimental data obtained using either optimal electrical stimulation parameters (*n* = 4 stimulations from two sagittal sections taken from two fish) or K+ stimulation (*n* = 2 stimulations from two fish)**.

**DA (μM)**	**HA (μM)**	**5-HT (μM)**	**Acid pH (pH units)**	**Basic pH (pH units)**	**EStim1 (*r*^2^)**	**Estim2 (*r*^2^)**	**Estim3 (*r*^2^)**	**Estim4 (*r*^2^)**	**K^+^ 10 s 1 (*r*^2^)**	**K^+^ 30 s 1 (*r*^2^)**	**K^+^ 10 s 2 (*r*^2^)**	**K^+^ 30 s 2 (*r*^2^)**
0.5	0	0	0	0	0.053	0.213	0.321	0.121	0.551	0.123	0.542	0.081
1	0	0	0	0	0.639	0.657	0.602	0.638	**0.78**	0.316	**0.836**	0.408
2	0	0	0	0	0.61	0.624	0.56	0.512	0.742	0.325	**0.81**	0.369
0	10	0	0	0	0.167	0.164	0.366	0.377	**0.75**	0.373	0.465	0.361
0	20	0	0	0	0.061	0.2	0.155	0.222	0.691	0.306	0.417	0.313
0	40	0	0	0	0.199	0.284	0.053	0.179	0.702	0.468	0.559	0.52
0	0	0.1	0	0	0.661	0.678	0.641	0.73	**0.758**	0.414	0.74	**0.612**
0	0	0.5	0	0	0.625	0.611	0.671	0.664	0.694	0.375	0.659	0.402
0	0	0.25	0	0	0.529	0.456	0.474	0.493	0.548	0.319	0.482	0.248
0	0	0	0	0.5	0.468	0.436	0.703	0.679	0.478	0.197	0.418	0.139
0	0	0	0	1	0.669	0.579	**0.814**	0.843	0.353	0.154	0.55	0.211
0	0	0	0	2	0.599	0.604	**0.834**	0.871	0.235	0.288	0.533	0.205
0	0	0	0.5	0	0.348	0.254	0.641	0.652	0.633	0.139	0.198	0.071
0	0	0	0.25	0	0.368	0.297	0.624	0.688	0.556	0.166	0.245	0.045
0	0	0	1	0	0.291	0.311	0.59	0.588	0.624	0.194	0.275	0.145
0	0	0	3.5	0	0.385	0.485	0.576	0.509	0.465	0.51	0.528	0.432
1	40	0	0	0	0.561	0.611	0.352	0.47	**0.833**	0.45	**0.76**	0.524
2	20	0	0	0	**0.814**	**0.787**	**0.771**	0.651	**0.857**	0.416	**0.866**	0.441
0	40	0.25	0	0	**0.71**	0.67	0.588	0.65	**0.762**	0.425	0.674	0.463
1	20	0.25	0	0	**0.776**	0.746	**0.784**	0.733	0.853	0.426	0.814	0.431
1	0	0	0	0.5	0.31	0.101	0.379	0.399	0.475	0.374	0.267	0.011
4	0	0	0	1	0.427	0.328	0.209	0.202	0.327	**0.508**	0.508	0.337
2	0	0	0.5	0	**0.833**	**0.755**	**0.832**	**0.868**	0.734	0.282	**0.752**	0.285
4	0	0	0.25	0	**0.841**	**0.802**	**0.818**	**0.873**	**0.781**	0.318	**0.819**	0.341
1	0	0	1	0	0.667	0.588	0.724	**0.801**	0.588	0.19	0.626	0.151
0	40	0	0	0.5	0.19	0.254	0.402	0.48	0.651	0.24	0.325	0.216
0	40	0	0	2	0.403	0.447	0.641	0.725	0.426	0.169	0.344	0.317
0	160	0	0.5	0	0.59	0.466	0.676	0.678	0.37	0.273	0.487	0.252
0	160	0	0.25	0	0.473	0.511	0.581	0.593	0.228	0.14	0.504	0.376
0	40	0	1	0	0.548	0.435	0.705	0.713	0.245	0.148	0.454	0.092
0	0	1	0	1	0.552	0.519	0.541	0.581	0.61	0.314	0.549	0.258
0	0	0.5	0	2	0.514	0.504	0.742	0.746	0.208	0.27	0.42	0.224
0	0	0.5	2	0	0.385	0.419	0.566	0.645	0.426	0.191	0.423	0.126
2	80	0	0	0.5	0.54	0.523	0.272	0.372	**0.759**	0.442	0.658	0.53
1	40	0	0	1	0.312	0.206	0.427	0.488	0.552	0.156	0.241	0.078
1	40	0	0.5	0	**0.795**	**0.755**	**0.75**	**0.804**	**0.826**	0.435	**0.808**	0.483
2	160	0	0.5	0	**0.845**	0.739	**0.84**	**0.853**	0.675	0.414	**0.75**	0.355
2	80	0	0.25	0	**0.846**	**0.833**	**0.863**	**0.877**	**0.777**	0.421	**0.824**	0.426
4	160	0	0.25	0	**0.845**	**0.806**	**0.863**	**0.878**	**0.752**	0.441	**0.801**	0.421
1	80	0	1	0	**0.752**	0.676	**0.799**	**0.831**	0.178	0.248	0.702	0.243
0	160	1	0.5	0	**0.75**	**0.727**	**0.836**	**0.813**	**0.762**	0.481	0.712	0.461
1	40	0.5	0	1	0.197	0.05	0.315	0.361	0.609	0.25	0.251	0.062

*Bold type indicates a significant r^2^-value, and bold red type indicates the highest r^2^-value for the example stimulation. Legend: DA, dopamine; HA, histamine; Estim, electrical stimulation; K^+^, high K^+^ aCSF stimulation*.

### Principal component analysis of electrical stimulation data

**Figure 8:**
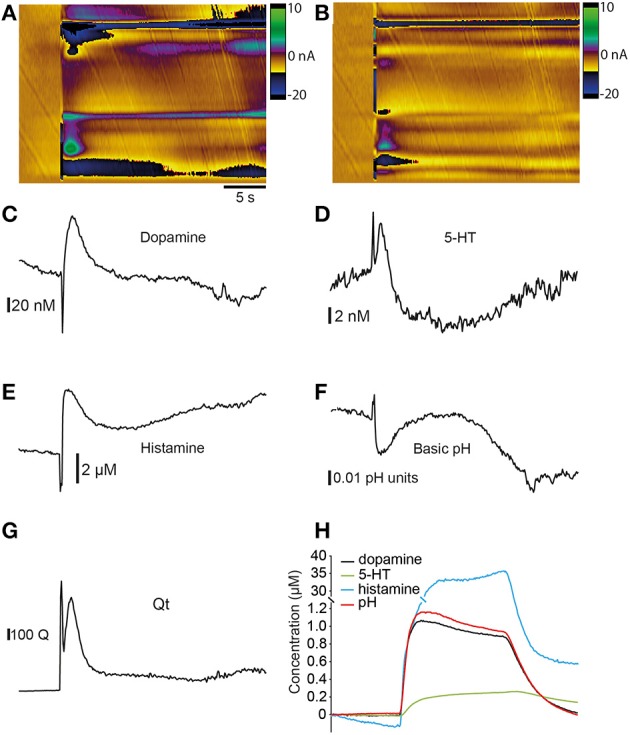
**Principal component analysis**. Principal component analysis (PCA) of data obtained from stimulation of zebrafish telencephalon. **(A)** Color plot showing current changes resulting from electrical stimulation. **(B)** Residual color plot showing changes not accounted for by the PCA model. **(C)** Time vs. concentration plot for dopamine obtained from PCA. **(D)** Time vs. concentration plot for 5-HT obtained from PCA. **(E)** Time vs. concentration plot for histamine obtained from PCA. **(F)** Time vs. pH units plot obtained from PCA. **(G)** Qt plot showing that data does not exceed the Q threshold of 686681. **(H)** Application of the model to a cyclic voltammogram representing a combination of 1 μM dopamine, 0.25 μM 5HT, 40 μM histamine and a pH shift of +1.0 unit measured in a flow cell.

We examined our data using principal component analysis to provide an estimate of actual neurotransmitter concentration in the brain and to determine the relative contribution of each neurotransmitter to the changes in current that we measured (Heien et al., 2005; Keithley and Wightman, 2011). We constructed training sets of voltammograms that included responses to both single neurotransmitters and mixtures of neurotransmitters in the flow cell. Full details of these training sets are provided in Table 1. We used data from electrical stimulation experiments (Figure 8A) for this analysis because recordings using high K^+^ aCSF did not fit the statistical model well, perhaps due to the prolonged time-course of the changes that can cause the baseline to drift. We obtained the best fit for our electrical stimulation data (i.e., the lowest residual values) when using a training set that included dopamine, 5-HT, histamine and both acidic- and basic pH shifts. The resulting concentration vs. time plots suggest that dopamine (Figure 8C), 5-HT (Figure 8D) and histamine (Figure 8E) are all likely to be present following electrical stimulation. Importantly, the resulting Qt plot did not pass the threshold of 686681 at any point (Figure 8G) suggesting that our training set fits the *in vitro* data well. The increase of dopamine is ~100 nM, 5-HT ~8.0 nM and the increase of histamine is ~8.0 μM. In addition, it appears that there is also an acidic pH shift of ~0.05 units (Figure 8F). To confirm that our PCA was accurate in its representation of type- and concentration- of analytes, we examined a combination of 0.25 μM 5HT, 1 μM dopamine, 40 μM histamine and an acidic pH shift of +1.0 unit obtained in the flow cell. This provided a highly accurate prediction of the concentration of each species (Figure 8H), suggesting that the training set was indeed appropriate for the main analysis.

### Pharmacological inhibition of dopamine reuptake

**Figure 9:**
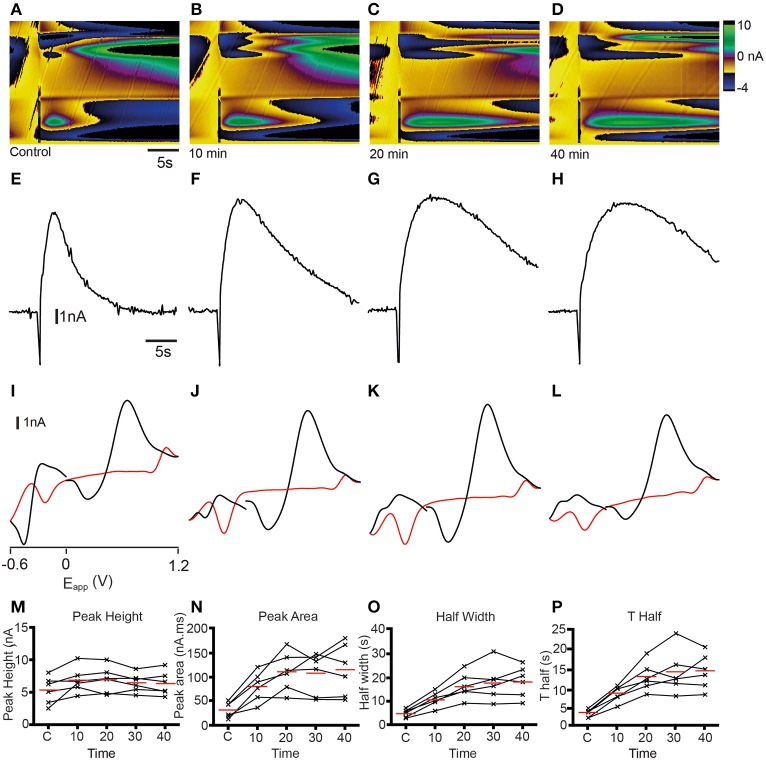
**Dopamine reuptake inhibition with cocaine modifies analyte release profile in the telencephalon. (A–D)** Color plots showing the time course (x-axis) of changes in current as a function of the applied waveform (y-axis) after a control stimulation **(A)** and 10 **(B)**, 20 **(C)**, and 40 **(D)** min after 10 μM cocaine administration. **(E–H)** Current vs. time plots showing the time course of changes in current after a control stimulation **(E)** and 10 **(F)**, 20 **(G)**, and 40 **(H)** min after 10 μM cocaine administration. **(I–L)** Cyclic voltammograms extracted immediately following onset of release with oxidation and reduction peaks becoming larger at ~ +0.65 V and ~ −0.25 V over time; control stimulation **(I)** and 10 **(J)**, 20 **(K),** and 40 **(L)** min after 10 μM cocaine administration. **(M–P)** Individual data points showing peak height **(M)**, peak area **(N)**, half width **(O),** and T half **(P)** for control stimulations (C1, C2, C3) or at the time points indicated.

**Figure 10:**
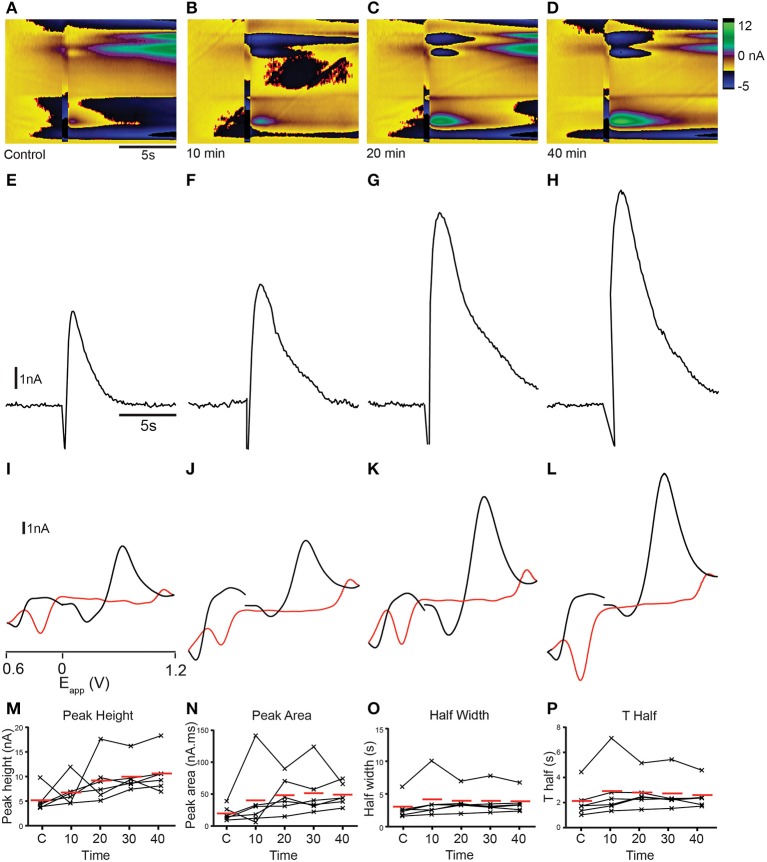
**Dopamine reuptake inhibition with GBR 12909 modifies analyte release profile in the telencephalon. (A–D)** Color plots showing the time course (x-axis) of changes in current as a function of the applied waveform (y-axis) after a control stimulation **(A)** and 10 **(B)**, 20 **(C)**, and 40 **(D)** min after 10 μM GBR 12909 administration. **(E–H)** Current vs. time plots showing the time course of changes in current after a control stimulation **(E)** and 10 **(F)**, 20 **(G),** and 40 **(H)** min after 10 μM GBR 12909 administration. **(I–L)** Cyclic voltammograms extracted immediately following onset of release, with oxidation and reduction peaks becoming larger at ~ +0.65 V and ~ −0.25 V over time; control stimulation **(I)** and 10 **(J)**, 20 **(K)**, and 40 **(L)** min after 10 μM GBR 12909 administration. Current vs. time plots showing the time course of changes in current after a control stimulation **(I)** and 10 **(J)**, 20 **(K)**, and 40 **(L)** min after 10 μM GBR 12909 administration. **(M–P)** Individual data points showing peak height **(M)**, peak area **(N)**, half width **(O)** and T half **(P)** for control stimulations (C1, C2, C3) or at the time points indicated.

The identity of analytes released during FSCV can be further confirmed by pharmacological validation (Dankoski and Wightman, 2013). The results of the CV match and PCA analyses suggest that dopamine is likely to be a major contributor to the changes in current that we measured. We further investigated this prediction by manipulating dopamine pharmacologically. We treated sagittal slices of the adult zebrafish brain with either cocaine, a non-selective monoamine reuptake inhibitor that has been shown to increase dopamine reuptake within the rodent nucleus accumbens, caudate putamen and substantia nigra (Jones et al., 1995a,b; Davidson et al., 2000; John and Jones, 2007a,b; España et al., 2008; Yorgason et al., 2011) or the selective long-acting dopamine reuptake inhibitor GBR 12909 (España et al., 2008; Esposti et al., 2013). Treatment with 10 μM cocaine produced an increase in current at ~ +0.6 V that appeared to be prolonged (Figures 9B–D) compared to controls (Figure 9A). Current vs. time plots showed peaks that become broader over time (Figures 9E–H) indicating a slowing down of reuptake kinetics. Comparison of cyclic voltammograms from these experiments further demonstrated that oxidation- and reduction peaks became more prominent at ~ +0.65 and ~ −0.25 V, respectively (Figures 9I–L) following cocaine application, with a shape that was more similar to the dopamine voltammogram obtained in the flow cell (Figure 2B). Current vs. time plots further illustrated this, as peaks became broader over time (Figures 9I–L) indicating a slowing down of reuptake kinetics. Moreover, there was a slight increase in the amplitude of peaks (Figures 9M,N), suggesting that cocaine may also affect dopamine release in the zebrafish telencephalon. We used One-Way repeated measures ANOVA tests followed by Dunnett's multiple comparisons tests with *p*-value adjustment to compare the average value of three control stimulations with four time-points following cocaine perfusion (*n* = 6 fish in each case). Non-parametric tests were used when the data were not normally distributed. There was a significant effect of cocaine on peak height [nA; *F*_(4, 20)_ = 4.91, *p* < 0.01]. *Post-hoc* Dunnett's tests revealed that peak height was significantly larger than control at 10 min (*p* < 0.005), 20 min (*p* < 0.005), and 30 min (*p* < 0.05) after cocaine perfusion, but not after 40 min (*p* > 0.05). Cocaine also had a significant effect on half width [s; *F*_(4, 20)_ = 23.14, *p* < 0.0001]. Half width was significantly increased 10 min (*p* < 0.01), 20 min (*p* < 0.0001), 30 min (*p* < 0.0001), and 40 min (*p* < 0.0001) after perfusion. There was also a significant effect on T Half [s; *F*_(4, 20)_ = 23.23, *p* < 0.0001]. T Half was significantly increased at 10 min (*p* = 0.005), 20 min, (*p* < 0.0001), 30 min (*p* < 0.0001), and 40 min (*p* < 0.0001). Cocaine also significantly influenced peak area [nA^*^s; *F*_(4, 20)_ = 19.17, *p* < 0.0001]. Dunnett's tests revealed peak area was significantly larger 10 min, (*p* < 0.005), 20 min (*p* < 0.0001), 30 min (*p* < 0.0001), and 40 min (*p* < 0.0001) following cocaine perfusion. Tau decay (s) was also significantly altered [$\chi_{\left(5\right)}^{2}=18.53$, *p* = 0.001 Friedman test]. *Post-hoc* tests showed that tau decay was significantly larger 20 min (*p* = 0.001), 30 min (*p* < 0.05), and 40 min (*p* < 0.005) following cocaine application (Figures 9M–P). Treatment with 10 μM of the more selective dopamine reuptake inhibitor GBR 12909 led to an increase in the amplitude and time course of current at ~ +0.6 V following electrical stimulation (Figures 10A–D). The related current vs. time plots show that peaks become larger and somewhat broader over time indicating an increase in release and possibly a slowing of reuptake kinetics as well (Figures 10E–H). Comparison of cyclic voltammograms from these experiments provided further evidence for an increase in the amplitude of release, as the peak around the oxidation potential for dopamine (~ +0.65 V) became considerably larger (Figures 10I–L). The effects of 10 μM GBR 12909 application were analyzed using non-parametric tests to account for significant deviation from normality. Friedman tests followed by *post-hoc* Dunn's multiple comparisons with *p*-value adjustment were used to compare the average value of three control stimulations with four time-points post GBR 12909 perfusion (*n* = 6 fish in each case). GBR 12909 had a significant effect on peak height [nA; $\chi_{\left(5\right)}^{2}$ = 16.93, *p* < 0.005]. Peak height was significantly increased after 30 min, (*p* < 0.05) and 40 min (*p* < 0.0001) drug application. GBR 12909 did not significantly alter half width (s; *p* = 0.0504), however there was a significant effect on T half [s; $\chi_{\left(5\right)}^{2}=10.4$, *p* < 0.05]. Dunn's tests revealed that T half was significantly increased after 20 min, (*p* < 0.05), 30 min (*p* < 0.05), and 40 min (*p* < 0.05). There was also a significant effect of GBR 12909 on peak area [nA^*^s; $\chi_{\left(5\right)}^{2}$ = 14.67, *p* < 0.01]. Peak area was significant increased after 20 min, (*p* < 0.05), 30 min, (*p* < 0.05), and 40 min, (*p* < 0.005) GBR 12909 perfusion (Figures 10M–P). GBR 12909 did not significantly alter tau decay (*p* > 0.05). Taken together, the combination of statistical analysis and pharmacological studies demonstrates that stimulation of the telencephalon evokes the release of dopamine, with possible release of histamine and a concomitant acidic change in pH as well.

## Discussion

In this study we have established a protocol to record the release of analytes in the zebrafish telencephalon by FSCV. We evoked neurotransmitter release by either bath application of high K^+^ aCSF or by electrical stimulation of local terminals. Neurotransmitters were detected and identified upon the basis of their oxidation and reduction profiles at the surface of a carbon fiber electrode. Using this method we obtained voltammograms that are significantly similar to the simultaneous measurement of dopamine and histamine coupled to a change in pH. We have also provided pharmacological validation that we have measured the release of dopamine. To the best of our knowledge, this study represents the first FSCV recordings in zebrafish, thus paving the way for neurochemical analysis of the fish brain.

### Comparison of slice preparation and method of stimulation

There are several advantages to using an *in vitro* slice preparation rather than recording from whole brains. Firstly, we were able to accurately place electrodes in a specific area, something that would be difficult to achieve in intact brains in the absence of a stereotaxic atlas. Our setup also gave us fine-control of environmental parameters (including temperature and pH) and obviated the need to use an anesthetic which could potentially alter the dynamics of neurotransmitter release (John and Jones, 2007a). We also compared stimulation of neurotransmitter release by either perfusion of high K^+^ aCSF or electrical stimulation. The results that we obtained with both methods were comparable once we had identified the best parameters for electrical stimulation (20 pulses with a pulse width of 4 ms, 60 Hz, 500 μA). The data that we obtained using low intensity stimulation was not very reproducible, and the oxidation peak was less prominent than in experiments using optimal parameters. In contrast to this, high intensity electrical- or K^+^ aCSF stimulation triggered a prolonged release profile that did not return to baseline. Electrical stimulation is ideal for examining local neurotransmitter release and could in theory be used to map neural circuits in the brain. However, we will have severed a large number of axon tracts when sectioning the brain meaning that the action of some endogenous control mechanisms (such inhibitory neurotransmitters) may have been disrupted (Dankoski and Wightman, 2013). The results from our slice preparation thus need to be interpreted with caution.

### Identity of neurotransmitters recorded in the telencephalon

A major challenge of FSCV is to characterize the analytes that are released following stimulation. Wightman and colleagues have suggested five criteria that can be used to identify endogenously released substances (Dankoski and Wightman, 2013): good correlation between voltammograms obtained during an experiment and standard data (e.g., when exposing electrodes to neurotransmitter solutions in a flow cell); independent verification of the presence of the neurotransmitter; precise anatomical positioning of the electrode in the region of interest; correct physiological release properties for the transmitter being measured; and pharmacological validation of each compound (Dankoski and Wightman, 2013). We have taken these criteria into account in this study. We compared our FSCV recordings in brain slices to template cyclic voltammograms generated in a flow cell using the CV match software (Figures 2, 6, 7). 5-HT, dopamine and histamine have already been shown to be present in the zebrafish forebrain by high pressure liquid chromatography and radioactive immunoassay (Norton et al., 2011; Buske and Gerlai, 2012). Furthermore, the position of our recording electrode was chosen based upon studies of the projection patterns of dopamine, 5-HT- and histamine neurons (Kaslin and Panula, 2001; Lillesaar et al., 2009). The voltammograms that we obtained from recordings in the telencephalon appear to represent a combination of more than one neurotransmitter. We do not appear to be measuring 5-HT since the voltammograms in Figure 3D do not show the characteristic reduction peak at 0 V (see Figure 3D). Our telencephalic recordings are most similar to an acidic (pH 6.95) solution of ~2 μM dopamine and 80 μM histamine recorded in the flow cell (Figure 7). We further validated this result by using the selective dopamine reuptake inhibitor GBR 12909 (Esposti et al., 2013). Application of GBR 12909 led to a significant alteration in peak amplitude, area and reuptake. We could not use the Michaelis-Menten kinetics to analyse this data, since the rate of dopamine reuptake in zebrafish (i.e., the rate of DOPAC formation in tissue slices) has not been calculated (Near et al., 1988) and it is unlikely that the stimulated release saturated reuptake, a prerequisite for Michaelis-Menten modeling. However, peak amplitude, area and reuptake parameters have already been used to examine FSCV data following pharmacological manipulation (Yorgason et al., 2011) demonstrating the validity of this approach.

### Characteristics of neurotransmitter release in the zebrafish telencephalon

The initial current vs. time plots that we recorded following high K^+^ aCSF stimulation of the telencephalon had two striking characteristics: a long release profile that lasted approximately 5 min (Figures 3C,E); and the presence of a second analyte that did not return to baseline (Figure 3F). Increasing the intensity of electrical stimulation also led to a longer time-course of release (Figure 4L). This result was surprising, since measurements of neurotransmitter release in the rodent brain typically only last for a few seconds (Hashemi et al., 2011). However, it could also indicate that a pH change has altered the background signal and thus inflated our measurements of current (Jones et al., 1994; Takmakov et al., 2010). Acidifying or alkalising pH shifts can occur in conjunction with neurotransmitter release and are an indicator of neural activity (Chesler, 2003; Venton et al., 2003; Takmakov et al., 2010). Therefore, care is needed to avoid confusing changes in pH and the release of neurotransmitters such as dopamine (Venton et al., 2003). In contrast to this, electrical stimulation with optimal parameters triggered a release with a shorter time-course that rapidly returned to baseline, making electrical stimulation with optimal parameters much more suitable for FSCV recordings in zebrafish. We obtained similar cyclic voltammograms by either electrically stimulating the adult zebrafish telencephalon or exposing an electrode in the flow cell to dopamine and histamine in the presence of an acidic pH shift (−0.5 pH units) (Figures 4E, 6E). This acidification fits within the normal physiological limit of pH changes (Chesler, 2003) and suggests that some of our current measurements in the adult zebrafish brain may have been influenced by alterations in pH concomitant with neurotransmitter release. Interestingly, the low concentration of 5-HT predicted by principal component analysis (PCA) suggests that this neurotransmitter may not contribute to the *in vitro* signal that we recorded; the trace level of 5-HT detected here could be an overestimation, caused by the overlapping oxidation potentials of 5-HT and dopamine, or the slight shift in oxidation potentials that can occur in tissue vs. flow cell recordings (Keithley and Wightman, 2011). Further experiments would be required to investigate this issue.

### Validation of FSCV data

We used CV match and PCA (Heien et al., 2005; Keithley and Wightman, 2011) to assess how well our training set fitted the data and to separate the constituent parts of the voltammograms elicited following stimulation of the telencephalon. The CV match programme calculated *r*^2^-values that exceeded the statistical threshold of 0.75 in each case (Figure 7), permitting us to conclude that it was highly likely we were measuring the release of dopamine, histamine and a change of -0.25 pH units. The CV match algorithm returns very conservative *r*^2^ estimates (Heien et al., 2003) meaning that we can be confident in the accuracy of this result. In contrast to this, we could not produce an electrochemical trace in the flow cell that surpassed the *r*^2^ threshold of 0.75 when compared to the change occurring ~30 s after high K^+^ aCSF stimulation (Figures 2F, 7F), perhaps due to distortion of the signal by high K^+^ levels and a change in pH (Threlfell and Cragg, 2007). We therefore cannot conclude anything about the analytes which may contribute to this change in current. We used PCA to partially account for our *in vitro* data by uploading a training set containing dopamine, 5-HT and histamine and both acidic- and basic pH changes (Figure 8). The quality of the PCA may be improved by using a biologically relevant training set rather than *in vitro* flow cell data. However, this was not achievable since initially we did not know which analytes we were detecting in the zebrafish brain. Future analyses could be improved by using green fluorescent protein (GFP) labeling to unambiguously identify neurons – for example, the ETvmat2:eGFP line labels all monoaminergic neurons in the brain (Wen et al., 2008).

### Neurochemical profile of the zebrafish brain

Stimulation of the zebrafish telencephalon appeared to trigger release of more than one neurotransmitter. FSCV has already been used to demonstrate the simultaneous release of 5-HT and histamine in the rat substantia nigra pars reticulata (Hashemi et al., 2011). Furthermore, the histamine H3 heteroreceptor can modulate the activity of many types of neurons (including dopamine and 5-HT neurons) thus permitting cross-talk between neurotransmitter systems (Haas et al., 2008). Zebrafish dopaminergic and histaminergic neurons project extensively throughout the brain (Eriksson et al., 1998; Kaslin and Panula, 2001) making it perhaps unsurprising that histamine and dopamine could be co-released in the telencephalon. The primary target of ascending histamine projections is the rostral medial dorsal telencephalon with some fibers innervating the caudal medial dorsal telencephalon (Eriksson et al., 1998). Therefore, the anatomical localization of histamine-positive fibers suggests that release of histamine could contribute to the change in current that we measured. Histamine plays a role in aggression, sleep, anxiety, locomotion and long-term memory in zebrafish (Peitsaro et al., 2000, 2003; Renier et al., 2007; Norton et al., 2011). The absence or scarcity of this neurotransmitter in the periphery (Eriksson et al., 1998) raises the possibility that histamine may have a more important role in the central nervous system of zebrafish than other vertebrates. Further work comparing the behavioral function of histamine in fish and other model organisms will be required to clarify this issue. Importantly, the protocol that we have established here can be used to examine the zebrafish dopaminergic system, including comparisons of dopamine reuptake in different brain areas and looking at the effect of pharmacological manipulations on neurotransmitter release. In addition, it will provide the basis for further characterization of zebrafish mutant lines that display alterations in behavior. FSCV thus represents another useful tool for in-depth characterization of the zebrafish brain.

## Author contributions

LJ, JM, AY, and WN designed the experiments. LJ conducted the experiments. WN wrote the first version of the manuscript. LJ, JM, AY, and WN improved the manuscript and approved the final version. We have no conflicts of interest to declare.

### Conflict of interest statement

The authors declare that the research was conducted in the absence of any commercial or financial relationships that could be construed as a potential conflict of interest.
